# Integrated Care Within New Zealand’s Specialist Mental Health and Addiction Services: Qualitative Research to Inform a New Model

**DOI:** 10.1007/s10597-026-01623-8

**Published:** 2026-04-10

**Authors:** Hannah King, Sarah Derrett, Emma H. Wyeth, Ruth Cunningham, Debbie Peterson

**Affiliations:** 1 Department of Preventive and Social Medicine, Ōtākou Whakaihu Waka | University of Otago, Dunedin, New Zealand; 2 Ngāi Tahu Māori Health Research Unit, Division of Health Sciences, Ōtākou Whakaihu Waka | University of Otago, Dunedin, New Zealand; 3 Ngāi Tahu Research Centre, Te Whare Wānanga o Waitaha | University of Canterbury, New Zealand, Christchurch, New Zealand; 4 Ngāi Tahu Māori Health Research Unit, Division of Health Sciences, Ōtākou Whakaihu Waka | University of Otago, Dunedin, New Zealand; 5 Department of Public Health, Ōtākou Whakaihu Waka | University of Otago, Wellington, New Zealand

**Keywords:** Mental health, Delivery of health care, integrated, New Zealand

## Abstract

Internationally, and within New Zealand (NZ), specialist mental health and addiction service-users (SMHAS-users) experience increased risks of comorbidities and premature mortality, and poorer health outcomes, compared to the general population. In particular, Indigenous Māori SMHAS-users face significant inequities compared to non-Māori. Integrated care has been found to improve outcomes by improving quality and coordination between services. This research aimed to develop a model of integrated care relevant to SMHAS-users in a NZ context, to inform the development of a questionnaire intended to assess SMHAS-users experiences of integrated care, support SMHAS to improve their services, and in turn, improve outcomes for SMHAS-users. Key informants working for, and/or with lived experience of, SMHAS were interviewed about existing integrated and people-centred care concepts. Barriers to integration were identified. The team met frequently to review and discuss coding, themes, and model development. Ten informants from across NZ were recruited, including five with lived experience of mental distress, and three who were Māori. Singer et al.,’s (*Medical Care Research and Review, 68*(1), 112–127, 2011) integrated patient care framework and the World Health Organization’s (2016) concept of people-centred care were found to be acceptable, although people-centred constructs were needed more clearly at a model’s core. SMHAS-user controlled funding and participation in multi-disciplinary team meetings were identified as key opportunities alongside other concepts such as cultural responsiveness, de-centralising services (to re-centralise people), and peer navigators. The resulting People-Centred Joined Up Care (PCJUC) model is proposed to convey a paradigm shift from a service-centric system, organised around the efficiencies and needs of services, to a people-centred system. Irrespective of the health workforce’s best intentions, the current service-centric orientation does not empower SMHAS-users. A people-centred model requires power structures to be inverted and the paramount authority of health professionals as the ‘decision-making experts’ to be challenged. Research is now underway exploring the use of a questionnaire developed to measure the concepts within the PCJUC model, to inform service improvements.

## Introduction

Internationally, and within New Zealand (NZ), specialist mental health and addiction service-users (SMHAS-users) experience increased risk of comorbidities and premature mortality compared to the general population (Cunningham et al., 2014; De Hert et al., 2011; Lockett et al., 2018; Te Pou o Te Whakaaro Nui, 2014; Wells et al., 2006). Socio-economic and lifestyle factors contribute to disparities in health and life expectancy (Richardson et al., 2019), as do inequities in access to, and quality of, health care (Collins et al., 2012; Hennekens et al., 2005; Lawrence & Kisely, 2010; Te Pou o Te Whakaaro Nui, 2014). Indigenous Māori SMHAS-users face significant inequities in burden of physical and mental illness, life expectancy, and poorer access and quality of care compared to non-Māori SMHAS-users (Cunningham et al., 2015; McLeod et al., 2017; Ministry of Health | Manatū Hauora, 2019; Russell, 2018; Wheeler et al., 2005). Factors contributing to these inequities include systemic racism, enduring effects of colonisation including reduced access to care, and socio-economic deprivation (Came, 2014; Graham & Masters-Awatere, 2020; Reid & Robson, 2007).

Whilst the NZ Government should improve health outcomes for all SMHAS-users in publicly funded services, it has specific obligations to address inequities experienced by Māori SMHAS-users in NZ due to its obligations under Te Tiriti o Waitangi (“Te Tiriti”). This is a treaty signed in 1840 between the British Crown and some Māori rangatira (chiefs) and is NZ’s founding document. Its purpose was to establish law and order amongst British settlers in NZ, whilst maintaining Māori tino rangatiratanga (absolute chiefly authority; self-determination), and guaranteeing Māori the same rights and privileges as British subjects (Orange, 2015). The Waitangi Tribunal, an independent statutory authority, has found that the Crown has breached Te Tiriti by failing to uphold the right to tino rangatiratanga, and not taking meaningful action to address entrenched health inequities faced by Māori (Waitangi Tribunal, 2023).

In NZ, the majority of SMHAS are publicly funded and are delivered in a range of inpatient, outpatient, and community settings. The private provision of SMHAS is very limited. The NZ health system has been undergoing significant reforms since 2022, and the resulting changes to the design and delivery of SMHAS continue to be worked through at local and regional levels (McGeorge & Every-Palmer, 2025).

A key driver of change in the NZ mental health system was the publication in 2018 of He Ara Oranga, the report of the Government Inquiry into Mental Health and Addiction (Patterson et al., 2018), which called for systemic changes in the design and delivery of mental health services in NZ. In response to recommendations from He Ara Oranga, there have also been changes to key legislation, including an initial amendment in 2021 to The Mental Health (Compulsory Assessment and Treatment) Act 1992 (“the Act”), ahead of a full repeal and replacement of the Act, which is underway (Mental Health Bill, 2024).

Integrated care improves the health and wellbeing outcomes for SMHAS-users by improving quality of care and strengthening coordination and continuity within and between services (Bellamy et al., 2016; Bradford et al., 2013). However, few studies have explicitly utilised any integrated care model or framework when investigating integrated care in mental health services, particularly in NZ (Richardson et al., 2019). ‘Integrated care’ is often inadequately defined but is considered synonymous with concepts such as ‘continuity of care’ (Haggerty et al., 2003; Richardson et al., 2019; Shaw et al., 2011) and ‘people-centred care’(69th World Health Assembly, 2016).

There is a need to understand how integrated (or ‘joined up’) care can best be conceptualised in NZ in a SMHAS context, particularly following the release of He Ara Oranga, which identified putting ‘people at the centre’ as key to transforming NZ’s mental health and addictions sector (Patterson et al., 2018).

This paper presents findings from a qualitative study which aimed to develop a model of integrated care relevant to NZ’s SMHAS, including concepts that matter to SMHAS-users. Given the significant inequities experienced by Māori SMHAS-users, and the NZ Government’s obligations to address these inequities within its SMHAS, the study sought to ensure the model is relevant and appropriate for both Māori and non-Māori SMHAS-users. However, the research was not conducted using Māori research methodology.

Longer term, the model was intended to inform the development of a questionnaire to assess SMHAS-users experiences of PCJUC in both inpatient and outpatient settings, so that SMHAS-users’ experiences of integrated care to help inform service improvements.

## Methods

Ethical approval was granted by the University of Otago Human Ethics (Health); H19/051. Data were collected via interviews with key informants following written consent. The objectives of the interviews were to:

1. Gain insights into what PCJUC means within mental health services in NZ, and what an ‘ideal’ PCJUC system might look like,
2. Identify barriers to achieving PCJUC for SMHAS-users in NZ.

Purposive and snowball sampling were used to identify potential informants from diverse SMHAS backgrounds (i.e. involved in SMHAS design, delivery, advocacy, or policy) (Palinkas, 2015), and/or with lived experience of SMHAS (Byrne, 2013). Potential key informants were initially identified from the professional and community networks of the academic supervisory team (SD, EHW, RC and DP) and student researcher (HK). Potential informants were invited to suggest, and provide an introduction, to other potential informants. Potential informants were initially contacted by email. Upon confirmation of interest from potential key informants, more detailed information about the research was shared before arranging interviews. Informants were offered a $30 (NZD) supermarket gift-card in appreciation of their participation.

Ten key informants with diverse professional and personal backgrounds from across NZ were recruited (see Table 1); five had lived experience of mental illness/distress, three were Māori, and one was Pacific. All were employed in the mental health and addictions sector in advocacy, policy, consumer advisory, or consultancy positions. Key informants came from a range of regions throughout NZ (four from the wider Wellington region, five from the Otago/Southland region, and one from the upper North Island).

Part way through recruitment, gaps in expertise were identified among the first five key informants. For example, whilst expertise in advocacy, peer-support, research, and policy were covered by these five key informants, expertise in service design and delivery, quality improvement, and integration between health and social services was needed. A further five key informants were recruited with expertise in these areas. Interviews (45–90 min) were conducted between July and September 2019 either face-to-face (*n* = 4), on the phone (*n* = 1), or via videoconference (*n* = 5); two informants elected to participate in a joint interview.

**Table 1:** Key informant background and expertise

Key informant	Background & expertise
1.	Māori consumer advisor with lived experience of mental distress and recovery, employed by a regional mental health and addictions services team. A former member of numerous national mental health and addictions advisory groups.
2.	Registered psychologist with experience in research, as well as national and international roles in mental health leadership.
3.	Māori senior leader for a Māori health and wellbeing organisation which has a particular focus on mental health and addictions.
4.	Consumer advisor with lived experience of mental distress, addiction, and recovery, working at a regional and national level. Also involved in research and teaching.
5.	Has held a range of leadership roles in national and international mental health and addictions organisations and human rights organisations, informed by their own lived experience of mental illness and recovery.
6.	Employed in a national advisory role with a focus on mental health and addictions quality improvement, informed by their own lived experience as a mental health service user as well as a health professional.
7.	A background in organisational leadership and the head of a Māori health service that provides a range of care to the community, including mental health and addictions services and other social and community services.
8 & 9	These key informants were interviewed together. Key informant 8 works with people experiencing mental distress and/or addiction, and key informant 9 has a background in medicine and youth development.
10.	This key informant did not give permission for their general expertise to be reported.

In this study, Singer et al.’s (2011) Framework for Measuring Integrated Patient Care (Friedberg et al., 2021; Singer et al., 2011), the World Health Organization’s (WHO) (69th World Health Assembly, 2016) and Goodwin’s (2014) concepts of people-centred care were explored with informants to understand how integrated care could be conceptualised in NZ. Singer’s framework, developed for a non-SMHAS context, comprises seven dimensions (Table 2).

Singer’s framework was selected because it has previously informed the development of a measure of integrated patient care among hospital patients – the Patient Perceptions of Integrated Care Survey (PPIC survey) – which was found to be reliable and valid in a United States context (Friedberg et al., 2021; Singer et al., 2013).

**Table 2:** Overview of the seven dimensions of the Singer Framework (Singer et al., 2011)

Care Coordination	Singer dimension 1	**Coordinated** **within** care team *“consistent with and informed by the care delivered by other team members [i.e. from the same care team]”* (Singer et al., 2011) (p119).
	Singer dimension 2	**Coordinated** **between** **care teams** Care that is *“consistent with and informed by the care delivered by other care teams”* (Singer et al., 2011) (p119).
	Singer dimension 3	**Coordinated between care teams and community resources** Care that is coordinated *“between patient care teams and support available through home and community resources”* (Singer et al., 2011) (p119).
	Singer dimension 4	**Continuous familiarity with the patient over time** Health professionals that are *“continuously familiar with the patient’s medical history… [including] familiarity with care he or she and others have provided to the patient in the past”* (Singer et al., 2011) (p119).
	Singer dimension 5	**Continuous proactive and responsive action between visits** “*Care team members reach out and respond to patients between visits”* (Singer et al., 2011) (p120), and *“patients can access care and information 24/7”* (Singer et al., 2011) (p120).
Patient-centredness	Singer dimension 6	**Patient-centred** Care in which *“providers consider the needs*,* preferences*,* values and capabilities of the patient*,* family members*,* and other care givers”* (Singer et al., 2011) (p121)
	Singer dimension 7	**Shared responsibility** *“…the patient*,* family members*,* and other caregivers are informed and engaged by providers in making health care decisions*,* providing care*,* maintaining health*,* and managing financial resources”* (Singer et al., 2011) (p121)

Singer’s framework had limitations in its application to this study, including that it was developed in a generic (i.e., not mental health-specific) and inpatient context in the United States. Whilst it explicitly identifies patients’ values, preferences, needs, beliefs and participation as vital to integrated care, the concept of ‘people-centred care’ has been defined as comprehensive, coordinated, collaborative, co-produced, continuous, empowering, holistic, and equitable (Goodwin, 2014; World Health Organization, 2015).

Consequently, people-centred care concepts were also explored, including a support networks and wider communities promoting the active participation of individuals, families and communities in the health system (69th World Health Assembly, 2016), valuing ‘the whole person’ as well as the holistic needs and goals of the person’s community (Goodwin, 2014).

Interviews, undertaken by HK, were semi-structured, utilising both conceptual and narrative interview styles (Kvale, 2009) to generate discussion and seek informants’ insights into the Singer framework and people-centred care concepts utilising a pragmatic methodological approach (Creswell & Poth, 2018). An interview topic guide was used to shape the discussion (Table 3). This flexible approach was taken to enable in-depth discussion and for key informants to focus on concepts they felt were most important. The interviews began with a ‘briefing’ (Kvale, 2009) to outline the purpose of the interview, the context of the research, and an overview of the discussion topics. Informants were asked to discuss the barriers they had experienced, or had seen SMHAS-users experience, in relation to integrated care. Additionally, Māori informants were asked to discuss specific barriers to integrated care faced by Māori SMHAS-users. Interviews were audio-recorded and detailed field notes taken. All informants except one gave permission for direct quotes to be used, either anonymously or identifiably, and were offered the opportunity to review their quotes before publication.

**Table 3:** Key informant interview guide in relation to the study objectives

Objective 1: Gain insights into what PCJUC means within mental health services in NZ, and what an ‘ideal’ PCJUC system might look like	
1. The Singer model	• The relevance and importance of each of the seven dimensions, in relation to developing a new PCJUC model for MHS-users in NZ • Contextual insight surrounding how each dimension is (or is not) operationalised in NZ • Any gaps and omissions in the framework that should be addressed in a new PCJUC model
2. ‘The ideal’ PCJUC experience	• The features of an ideal experience of PCJUC within and between services for MHS-users, in relation to the development of a new PCJUC model for MHS-users in NZ
Objective 2: Identify barriers to achieving PCJUC for SMHAS-users in NZ	
3. Barriers:	• Faced by MHS-users in accessing primary care, other (non-mental health) secondary/tertiary services, and/or social and community services • To achieving each of the Singer framework’s seven dimensions • Points in the care pathway where PCJUC breaks down

After listening to audio-recordings, the focus for qualitative analyses were the detailed field notes. These were deductively coded (by HK; reviewed by SD) in relation to the Singer framework’s seven dimensions and the people-centred concepts. Inductive coding of ‘new concept’ codes was undertaken for concepts which either ‘expanded’ the Singer and WHO concepts or were ‘new’ concepts raised by informants (Tracy, 2024). The wider team met frequently to review and discuss the coding and model development. Audio-recordings were reviewed to supplement gaps in field notes and for illustrative quotes.

## Results

**Fig. 1:**
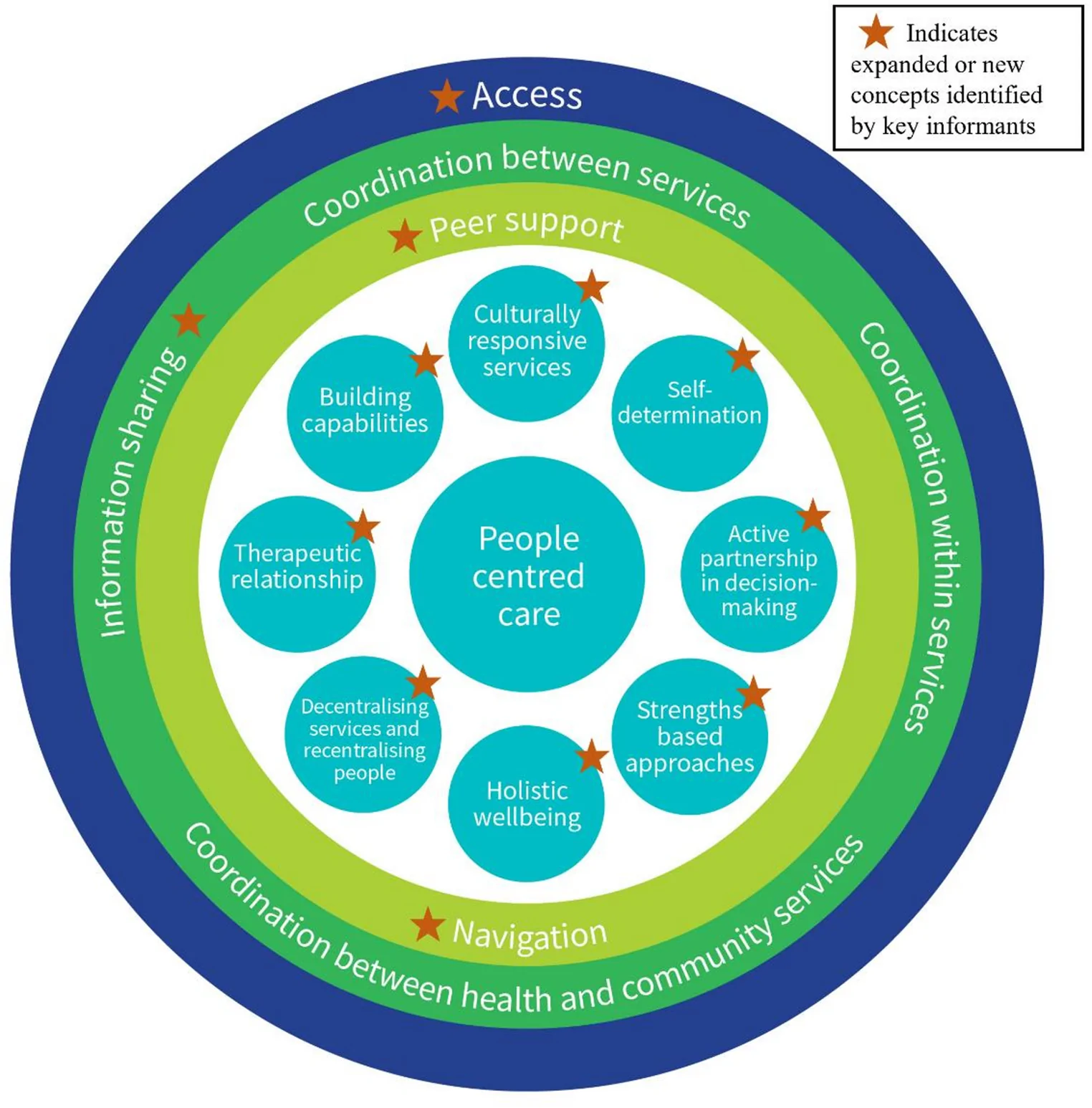
The people-centred joined up care (PCJUC) model

The key informants shared insights into each of the seven domains of the Singer Framework, identified concepts of integrated and people-centred care for SMHAS-users that were not sufficiently defined by Singer’s framework, and discussed barriers to achieving these concepts. These are presented below, culminating in the development of the People-Centred Joined Up Care model in Fig. 1.

Many informants found the term ‘integrated care’ unsatisfactory, despite finding integration concepts (such as coordination and continuity) important and engaging.

The first dimension in Singer’s framework describes care that is *“consistent with and informed by the care delivered by other team members (i.e. from the same care team)”* (Singer et al., 2011: page 119). Despite unilaterally agreeing intra-team coordination is key to integration, some key informants explained that coordination within teams is hindered by operational constraints, such as a lack of time. Key informants with clinical backgrounds generally were more optimistic about the success of within team coordination. In contrast, those with lived experience of mental illness tended to share negative experiences of inadequate coordination within teams, needing to explain their reasons for seeking care to multiple team members in a single visit.

Singer’s second dimension describes care that is *“consistent with and informed by the care delivered by other care teams”* (Singer et al., 2011: page 119). Informants agreed that SMHAS-users experience inconsistent and poor coordination across teams, particularly between primary care and SMHAS, leading to poor outcomes. For example, some key informants discussed a scenario described as commonplace where inpatient SMHAS could change a person’s care plan without consulting/informing the person’s outpatient care team, and then discharging the person back into the care of outpatient services. This was attributed to siloed, under-resourced, and over-worked services, lacking the time and processes necessary to share information:

*“That’s always been one of the things that…we’ve never been able to get…That [a person’s] psychiatrist stays the same no matter where they are [receiving care]…the same thing applies with the social worker and the nurse within the community team…that is probably the biggest issue with coordinated care between services*,* is the inconsistency of the staff working with the person.”* (Informant 1).

The third Singer dimension is care coordinated *“between patient care teams and support available through home and community resources”* (Singer et al., 2011: page 119). Informants agreed this would be beneficial for SMHAS-users and their whānau (extended family, kin network), and that lack of established linkages and processes for information sharing and referrals between SMHAS and community services creates challenges to coordinated care. Some key informants suggested inadequate knowledge amongst clinicians about the range of social/community supports available, as well as knowing how to connect people with those services. The key informants felt that an overly clinical focus with respect to recovery amongst health care professionals could limit their insight into broader holistic wellbeing needs of a person, and the resources available to support them.

The fourth dimension notes health professionals should be *“continuously familiar with the patient’s medical history…[including] familiarity with care…provided to the patient in the past”* (Singer et al., 2011: page 119). Some informants questioned whether a peer support worker or navigator should be the central figure of continuous familiarity, rather than a clinician. It was also suggested that it could be the person’s ‘usual care team’ members (e.g., General Practitioner (GP), community mental health worker or social worker), rather than those working in SMHAS.

Additionally, informants reflected upon the broader role of ‘information’ and proposed a new concept (labelled self-determination) that ensures the agency of SMHAS-users and their whānau in determining what information is shared, and with whom:

*“Who owns the data?…Wouldn’t that be interesting*,* if there were families that said*,* ‘Look – we’re happy for [these different agencies] to work with us*,* however*,* we will determine how our files are shared*,* what [service providers] write in them*,* and how they are [used in] conversations and planning for solutions going forward.’ I mean that would be a totally different power shift.”* (Informant 3).

Some informants noted that SMHAS-users could be justifiably concerned that if, for example, their oncologist or cardiologist was aware of their mental illness diagnosis, they might be treated dismissively and could receive poorer quality care:

*“Some of it honestly comes down to [the fact] we don’t value the lives of people with serious mental illness as much as other people. So*,* we’re not going to go to such efforts to make sure they live a long life*,* I mean I think there’s an underlying pessimistic belief about people [with serious mental illness] that pervades some of this lack of integration.”* (Informant 2).

A single SMHAS-user controlled electronic health record was proposed, allowing all services the person engages with to access their record with their permission. This would restore the SMHAS-user’s sovereignty over their own information, alleviating privacy concerns which was noted as needlessly stifling past attempts to establish information sharing processes between services.

Singer’s fifth dimension has two components: (1) *“Care team members reach out and respond to patients between visits”*, and *(2) “patients can access care and information 24/7”* (Singer et al., 2011: page 120). Informants agreed that service provider proactivity is positive, and that contacting SMHAS-users to ‘check in’ could be a welcome step in building a therapeutic relationship. However, some stressed this could equally be viewed by SMHAS-users as overly intrusive.

Ensuring services are accessible and responsive to SMHAS-users between scheduled appointments and outside ‘office hours’ was agreed to be important, with many informants identifying the lack of after-hours and weekend community SMHAS as a problem. Singer’s framework recognises ‘access’ as a component of integrated care, however, key informants expanded upon this to identify four specific barriers to access: cost, clinical criteria/thresholds to access, long wait times, and service accessibility (location, transport and time). One informant explained that as a person moves along the continuum of ‘well’ to ‘unwell’, there are structural barriers to accessing the range of supports, which they described as *“doors shutting behind them”* (Informant 2). For example, once someone is formally admitted to tertiary SMHAS, they often stop qualifying for community-based services such as peer-support or community-based counselling. The cause was elusive, but a lack of funding – restricting the number of people accessing the service – was offered as a potential explanation. Additionally, the lack of support available to people experiencing distress overnight and during weekends was seen as a particularly concerning gap. This was noted as a particular issue for Māori in some regions, where insufficient funding meant Māori services only operated during week-day business hours.

Singer’s final two dimensions are *“providers consider the needs*,* preferences*,* values and capabilities of the patient*,* family members*,* and other care givers*”, and *“…the patient*,* family members*,* and other caregivers are informed and engaged by providers in making health care decisions*,* providing care*,* maintaining health*,* and managing financial resources”* (Singer et al., 2011: page 121). Given the WHO’s emphasis on people-centred, rather than person-centred care, both dimensions are referred to in this analysis as ‘people-centred’ care (Goodwin, 2014; World Health Organization, 2015). Informants regarded these dimensions as inseparable. People-centredness was also linked by informants to the expanded concept of ‘self-determination’ mentioned above.

The potential for SMHAS-user controlled individualised funding was raised as a key opportunity for self-determination and active partnership. However, currently, only those with complex and ongoing needs can access individualised funding (Te Pou o Te Whakaaro Nui, 2014). Active participation of SMHAS-users and whānau in multi-disciplinary team (MDT) meetings was also highlighted as critical for self-determination and active partnership. In MDT meetings, health professionals from across different care teams (and sometimes social services) meet to discuss and make changes to the SMHAS-users care plan. Accordingly, MDT meetings are an excellent example of Singer dimensions 1–3. However, informants explained it is rare for SMHAS-users to be included in MDT meetings:

*“A person had all of these decisions made about them*,* without them*,* and they [the decisions] were things that the person did not want.”* (Informant 1).

It was important that SMHAS-user (and whānau) participation in MDT meetings must be supported appropriately to ensure they are not *“…set up to fail”.*

Informants advocated for systems to support SMHAS-users in making decisions, rather than making decisions for them. Other expanded concepts within the umbrella of ‘people-centredness’ were cultural responsiveness, de-centralising services (to re-centralise people), building capabilities, using strengths-based approaches, support for holistic wellbeing, and developing therapeutic relationships.

All informants identified respecting and responding to SMHAS-users’ cultural beliefs and practices as central to people-centred care. Cultural safety and responsiveness were regarded as necessary to building a holistic understanding of the SMHAS-user, rather than solely through the lens of their mental health diagnosis. Furthermore, this information would support planning which could, for Māori, include traditional Māori healing practices and activities that build cultural identity and foster connection with their community. These ideas link more broadly to the importance of deconstructing power structures within health and social services that privilege non-Māori worldviews surrounding ‘what is best’ for SMHAS-users:

*“I think the challenge in New Zealand still is: who says what works? And who says what evidence is the right evidence to prove the outcomes [we’re working towards]?”* (Informant 3).

This challenges an entrenched power dynamic existing between health care professionals and SMHAS-users, and the assumption that the professional ‘knows best’; prioritising instead the SMHAS-user’s voice in decision-making.

Informants described a deficit-oriented approach to care in NZ as engrained and stemming from a biomedical model of care, which is primarily concerned with an individual’s physical condition and its biological drivers (Cram et al., 2003; Elers, 2014; Wilson & Barton, 2012). Through its dominance, the biomedical model has been described as dismissive of Māori approaches to health, which are conversely holistic, recognise the role of mental and spiritual wellbeing, and focused on the interconnection of people, their whānau and communities, and their wider environment (Masters-Awatere et al., 2020). The holistic nature of wellbeing was identified as central to any understanding of people-centredness. Key informants also discussed the importance of people-centred care from a Te Ao Māori viewpoint; many citing ‘Te Whare Tapa Whā’ (Durie, 1985), which is widely used as a Māori model for understanding the holistic nature of health. Accordingly, and linking back to dimension three, truly people-centred care must include services that work beyond a health context, such as services operating within the housing, income support, and education sectors.

Building and maintaining a trusting and open therapeutic relationship between care team members, SMHAS-users, and their whānau was a new concept identified by many of the key informants as central to people-centredness. The concept of the therapeutic relationship moves beyond Singer’s fourth dimension to the interpersonal relationship between the SMHAS-user and providers. One informant described this as a ‘therapeutic alliance’ and could also involve whānau.

New components important for integrated care were raised which did not align directly with dimensions in Singer’s framework: peer support and navigation. Access to peer support workers (people with lived experience of mental distress and recovery) was identified as useful by many informants. Benefits included the potential of peer support workers to provide a non-clinical and relationally focused source of support, and the ability of peer support workers to identify relevant resources/services. It was proposed that peer support workers could also be involved in MDT meetings, as both a care team member and support person for the SMHAS-user.

There are regional disparities in access to peer support networks in NZ. It was suggested that poor attitudes among service providers towards the value and expertise of lived experience contributed to the under-development of the peer support workforce:

*“There’s always this distinction or determination between what’s qualified and what’s not qualified…What we need to shift to is: a way of being where actually there are a number of different wounded healers within communities and families who just need a little bit of support – and employment.”* (Informant 3).

A related, but distinct, concept raised was the importance of navigators to guide SMHAS-users and whānau along their recovery by linking them with services. This coordination function links to Singer’s dimensions two and three.

Figure 1 presents the People-Centred Joined Up Care (PCJUC) model which was developed from interview findings and informed by Singer’s framework and people-centred care concepts. Asterisked items are expanded or new concepts – beyond those already presented in Singer’s Framework – which were identified by the key informants as necessary for achieving integrated care in the NZ context.

## Discussion

Singer’s integrated care framework and the WHO concept of people-centred care were used as conceptual foundations for developing a working model of PCJUC for SMHAS-users in NZ (Singer et al., 2011). Overall, key informants supported Singer’s framework; agreeing that the seven dimensions are important facilitators of good care. However, key informants felt that the term ‘integrated care’ was insufficient, and if the model was to reflect what matters to SMHAS-users, the people-centred construct must be at the model’s core rather than periphery, and that a circular model (rather than linear) appears necessary for NZ (Fig. 1). Importantly, the findings of He Ara Oranga also emphasise that true transformation of NZ’s services requires people to be at the centre of the system (Patterson et al., 2018).

Informants also identified aspects of people-centred care that expanded upon the Singer framework, including self-determination, culturally responsive services, strengths-based approaches, building capabilities, and the therapeutic relationship. In addition to these expansions, ‘new’ concepts (peer support and navigators) were identified.

This new people-centred model engages people as active and equal partners in decision-making, where SMHAS-users and their whānau must be trusted, respected, and supported to make decisions based on their needs and aspirations for wellbeing. It is founded upon respectful therapeutic relationships, considers people’s holistic wellbeing (including culturally responsive services), is strengths-based, and builds on people’s capabilities for self-advocacy and self-management. Wrapping around these people-centred constructs are relationally-focussed peer-support and navigation services. Surrounding these constructs is ‘access’; positioned as the outermost ring of the new model because obtaining access to services pre-empts any experience of PCJUC.

Informants identified strengths-based approaches as an expansion of Singer’s framework, explaining that these are the embodiment of people-centredness. Singer’s framework discusses care that ‘considers’ people’s capabilities. However, key informants emphasised building SMHAS-users’ (alongside their whānau/family/friends) capability to self-manage and self-advocate, as crucial to a people-centred system, and to *‘moving from a service-centric system to a people-centred system’.*

Singer’s framework describes outcomes of effective therapeutic relationships (e.g. *“doctors know all of my important medical information”*). However, informants described open and trusting therapeutic relationships as facilitating many aspects of PCJUC, as well as a feature of PCJUC itself. People are relational, and relationships and connections are key to wellbeing (Durie, 1985); therefore people-centred care is also inherently relational. Including the therapeutic relationship construct within the new PCJUC model emphasises the centrality of SMHAS-users’ relationships with care workers to their overall experience of PCJUC.

Two new PCJUC concepts (peer-support and navigation) identified by informants are highly beneficial therapeutic tools for people experiencing mental illness/distress (Repper & Carter, 2011). Through a lens of decentralising services and recentralising people, peer-support was viewed as having potential to democratise services by delivering services that are by people with lived-experience, for people with lived-experience. This does not negate the need for access to specialist clinical support, but rather, emphasises the value of lived experience and peer support expertise as part of a multi-disciplinary team. It is also essential that peer-support workforces are adequately supported, trained, and resourced in order to realise the workforces’ full potential (Te Pou o Te Whakaaro Nui, 2020).

Whereas Singer’s framework focuses on coordinating and joining-up services, Informants identified navigation as one possible solution to the complexity of moving within and between services. For example, in NZ Whānau Ora Kaiārahi (navigators) have been shown to contribute to improved outcomes for SMHAS-users, improved whānau engagement with services (Stewart et al., 2012), and development of strong therapeutic relationships with SMHAS-users (Hannigan et al., 2018). Within the PCJUC model, navigation is placed in-between people-centred care and coordinated care.

Beyond the domains of Singer’s framework, a range of challenges for implementing PCJUC were raised by key informants, including limited time and resources within SMHAS, and barriers to sharing information between service providers. Many of these challenges are consistent across the wider health system in NZ and require system-level interventions (Waitangi Tribunal, 2023). Accordingly, whilst the PCJUC model conceptualises an ‘ideal’ PCJUC experience in SMHAS in NZ, the ability of services to respond to the model is, and will continue to be, significantly constrained until the wider system settings and funding are adjusted to enable PCJUC.

That said, there are a range of potential drivers of PCJUC that already feature within the NZ health and disability system. One example is the “Code of expectations for health entities’ engagement with consumers and whānau”, which is legislated under the Pae Ora Act 2022, and requires health entities to work with service-users, whānau and wider communities in the design and delivery of services (Health Quality and Safety Commission | Te Tāhū Hauora, 2022). Another example is the inclusion of specific actions within Te Pae Tata – the interim NZ Health Plan 2022 to improve access, choice, and flexible models of care for people living with mental distress, including Kaupapa Māori and Pacific services (Health New Zealand | Te Whatu Ora, 2025; Te Whatu Ora & Te Aka Whai Ora, 2022). There are also examples of existing initiatives to improve integration of services for people experiencing mental distress and access to support, such as the expansion of health improvement practitioner (HIP) and health coach roles in primary care, for example using the Te Tumu Waiora model (Te Tumu Waiora, n.d.). Whilst these specific roles were not discussed with key informants, they appear to deliver navigator-like functions to connect with other health social services, as well as providing direct advice and support for service-users.

Within NZ, publicly funded health services are obliged to uphold Te Tiriti o Waitangi and improve outcomes for Māori. Kaupapa Māori models are fundamental to improving care and outcomes for tangata whaiora Māori (people seeking wellness) (Waitangi Tribunal, 2023) and there has been (and continues to be) extensive work led by Iwi and Kaupapa Māori organisations, clinicians, and researchers to improve access to care and outcomes for Māori (Rangihuna et al., 2018). The PCJUC model is not intended to articulate what PCJUC means from a Māori perspective. Rather, it was designed for ‘mainstream’ specialist MHAS in NZ with the intent to be consistent with giving effect to Te Tiriti, whilst acknowledging that tangata whaiora Māori have a fundamental right to accessing Māori-led and delivered services (Waitangi Tribunal, 2023).

The PCJUC model could support mainstream services to uphold their Te Tiriti obligations in the provision of care to Māori SMHAS-users firstly through its focus on self-determination, echoing their fundamental right to tino rangatiratanga, as guaranteed under Te Tiriti (Waitangi Tribunal, 2023; Wyeth et al., 2010) Secondly, equity, also guaranteed under Te Tiriti, commits health services to achieving equitable outcomes for Māori (Reid & Robson, 2007; Wyeth et al., 2010). Given the gains in health and wellbeing outcomes possible through integrated care and people-centred practices (Bellamy et al., 2016; Bradford et al., 2013), embedding PCJUC concepts within SMHAS may help support the re-orientation of services around Māori SMHAS-users and could contribute to improving outcomes and addressing inequities faced by Māori SMHAS-users. Further research is needed to assess the potential of the PCJUC model in this regard. Finally, the PCJUC model includes dimensions of ‘culturally response services’ and ‘holistic wellbeing’, which could give effect to Te Tiriti by providing care that protects Māori values, customs, faiths, and spirituality.

Key informants had a wide range of expertise in the mental health and addictions sector, including leadership roles. However, our study was limited in that it is based on a small sample size of people (*n* = 10), and there were gaps in the expertise and experience of those interviewed. For example, people in peer support roles were not interviewed, and we did not ask whether any of the key informants were current SMHAS-users – although the expertise of key informants with lived experience of mental distress and/or addiction and who had previously accessed SMHAS services were specifically sought.

The model could have been strengthened with a group discussion amongst the key informants to test, refine, and elaborate the concepts they discussed in their separate interviews. Throughout the interviews, there were limited instances of differing or opposing views, one example being that key informants with clinical backgrounds tended to be more positive about the success of within team coordination compared to those with lived experience of mental illness. Bringing the key informants together to discuss these issues may have presented the opportunity to draw out further points of differing perspectives. However, this was challenged by both time constraints and because some key informants wanted to remain anonymous.

Informants also reported a range of ethnicities, including three Māori, however only one person was Pacific; further research specifically exploring joined up care from Māori and Pacific perspectives is warranted. A strength of our study was that half the informants reported lived experience of mental illness/distress which was important for helping ensure the development of a meaningful and appropriate PCJUC model. These key informants were therefore able to draw on both their own lived experiences of using mental health and addiction services, and their professional experiences working in the sector, when discussing PCJUC concepts.

## Conclusion and Relevance for Clinical Practice

The new PCJUC model conveys a potential paradigm shift from a service-centric system, organised around efficiencies and needs of services, to a people-centred system. Irrespective of health workers’ best intentions, the current service-centric system does not empower SMHAS-users and their whānau/support networks. Transitioning to a people-centred model of care requires prevailing power structures to be inverted and the paramount authority of health professionals as the ‘decision-making experts’ to be challenged.

The concepts articulated in the new PCJUC model align well with He Ara Oranga findings, such as the importance of active partnership in decision-making, of giving weight and value to patient and whānau voices, whānau involvement in decision-making, and joining up services and care (Patterson et al., 2018). It is hoped this model may be of use to health professionals, SMHAS-users and policy-makers as NZ continues to go through significant health reform.

Research is currently underway exploring the use of a questionnaire developed to measure integrated care according to the concepts within PCJUC. This tool is intended to measure SMHAS-users’ experiences of PCJUC, identify gaps in PCJUC, measure progress in delivering improved PCJUC over time, inform quality improvement for SMHAS, and ultimately, to lead to more equitable outcomes for SMHAS-users in NZ.

## Data Availability

Data cannot be shared openly to protect participant privacy, however quotes presented within the paper have received participant approval for sharing.

## Glossary of Māori terms

Iwi: extended kinship group, tribe, nation, people, nationality, race - often refers to a large group of people descended from a common ancestor and associated with a distinct territory.
Kaupapa Māori: Māori approaches, ideologies
Kaiārahi: Navigators
Tāngata whaiora: ‘tāngata whaiora’ translates to ‘people seeking wellness’ and is a term used to describe people who are seeking wellness from mental distress or illness.
Te ao Māori: the Māori World, a holistic worldview encompassing Māori cultural concepts and practices
Te Tiriti o Waitangi: The Treaty of Waitangi
Tino rangatiratanga: self-determination, chiefly authority
Whānau: family, extended family, close connections
